# Toll-like receptors and integrins crosstalk

**DOI:** 10.3389/fimmu.2024.1403764

**Published:** 2024-06-10

**Authors:** Fahd Alhamdan, Ganchimeg Bayarsaikhan, Koichi Yuki

**Affiliations:** ^1^ Department of Anesthesiology, Critical Care and Pain Medicine, Cardiac Anesthesia, Boston Children’s Hospital, Boston, MA, United States; ^2^ Department of Anesthesia and Immunology, Harvard Medical School, Boston, MA, United States; ^3^ Broad Institute of MIT and Harvard, Cambridge, MA, United States

**Keywords:** toll-like receptor, β1 integrin, β2 integrin, αV integrin, crosstalk

## Abstract

Immune system recognizes invading microbes at both pathogen and antigen levels. Toll-like receptors (TLRs) play a key role in the first-line defense against pathogens. Major functions of TLRs include cytokine and chemokine production. TLRs share common downstream signaling pathways with other receptors. The crosstalk revolving around TLRs is rather significant and complex, underscoring the intricate nature of immune system. The profiles of produced cytokines and chemokines via TLRs can be affected by other receptors. Integrins are critical heterodimeric adhesion molecules expressed on many different cells. There are studies describing synergetic or inhibitory interplay between TLRs and integrins. Thus, we reviewed the crosstalk between TLRs and integrins. Understanding the nature of the crosstalk could allow us to modulate TLR functions via integrins.

## Introduction

Immune cells are mounted with a number of pattern recognition receptors (PRRs) that recognize foreign pathogens. Microbial components are main targets for host immune cells to use for the recognition of microbes, and Toll-like receptors (TLRs) are one of major PRRs and evolutionarily ancient mediators for innate host defense (1, 2). Other PRRs include RIG-I-like receptors (RLRs), Nod-like receptors (NLRs), and C-type lectin receptors (CLRs) (3). So far 10 human TLRs (TLR1-TLR10) and 12 mouse TLRs (TLR1–9, TLR11–13) are identified (4). They are expressed on the plasma membrane or the endocytic vesicles.

Among all the TLRs, TLR4 has been studied most extensively. TLR4 mainly recognizes lipopolysaccharide (LPS) of Gram-negative bacteria (5, 6). To demonstrate its function, TLR4 binds to adaptor protein MD-2 to form TLR4-MD-2 complex (7). TLR4-MD-2 complex binds to LPS, then forms a dimer to activate intracellular signaling cascade. Other TLRs also form dimers (homodimer or heterodimer) to be functional. TLR2 recognizes peptidoglycan, lipopeptide, and lipoprotein of Gram-positive bacteria in concert with TLR1 or TLR6 (8, 9). TLR3 recognizes double-stranded RNA (dsRNA) (10). TLR5 recognizes bacterial flagellin (11). TLR7 and TLR8 recognize single-stranded RNA (ssRNA) (12–14). TLR9 recognizes bacterial and viral CpG DNA motifs (15, 16). The recognition of microbial pathogens by TLRs induces the activation of intracellular signaling pathways, resulting in the production of inflammatory cytokines, type I interferon, and chemokines. TLRs also induce the upregulation of costimulatory molecules on dendritic cells (DCs) (17). TLR10 is the latest human TLR to be discovered, and its ligand is still unclear (18). In contrast to TLR1–9, TLR10 demonstrates anti-inflammatory response (19, 20). While it is known to respond to influenza virus infection (21), this TLR still requires more extensive work in the future.

In addition to recognizing exogenous ligands derived from microbes, TLRs interact with endogenous molecules released from damaged tissues or dead cells (22). For example, high mobility group box 1 (HMGB1) is a nonhistone nuclear protein (23) and can bind to TLR2, TLR4, and TL9. The list of ligands for each TLR is listed in **Table 1**. The location of each TLR is also shown.

**Table 1:** List of TLRs, their location and ligands.

TLRs	Ligands	Expression
TLR2 TLR3 TLR4 TLR5 TLR7 TLR8 TLR9	Triacyl lipopeptide (24), peptidoglycan, lipopeptide, lipoprotein, zymosan (25), HMGB1 (26), HSP60 (27), HSP70 (28), hyaluronan (29) dsRNA (10), mRNA (30) LPS (5), HMGB1, HSP60, HSP70, hyaluronan flagellin (11) ssRNA (31), siRNA (32) ssRNA (31), siRNA (32) unmethylated CpG (15), HMGB1 (33), DNA (34)	Plasma membrane Endosome Plasma membrane Plasma membrane Endosome Endosome Endosome

The production of pro-inflammatory mediators such as cytokines and chemokines is one of the major TLR functions. Those inflammatory mediators would help regulating the immune system (35). For example, TLR2 and TLR4 are recognized by various ligands (**Table 1**). However, inflammatory response by different TLR2 ligands may not be the same. The involvement of non TLR receptors can provide a more tailored, specific response to TLRs. Integrins are critical adhesion molecules involved in many biological processes and play an important role in TLR crosstalk. Thus, we will first describe TLR signaling pathways. Then we will examine the role of integrins as regulators of TLR functions.

## TLRs signaling pathways

**Figure 1:**
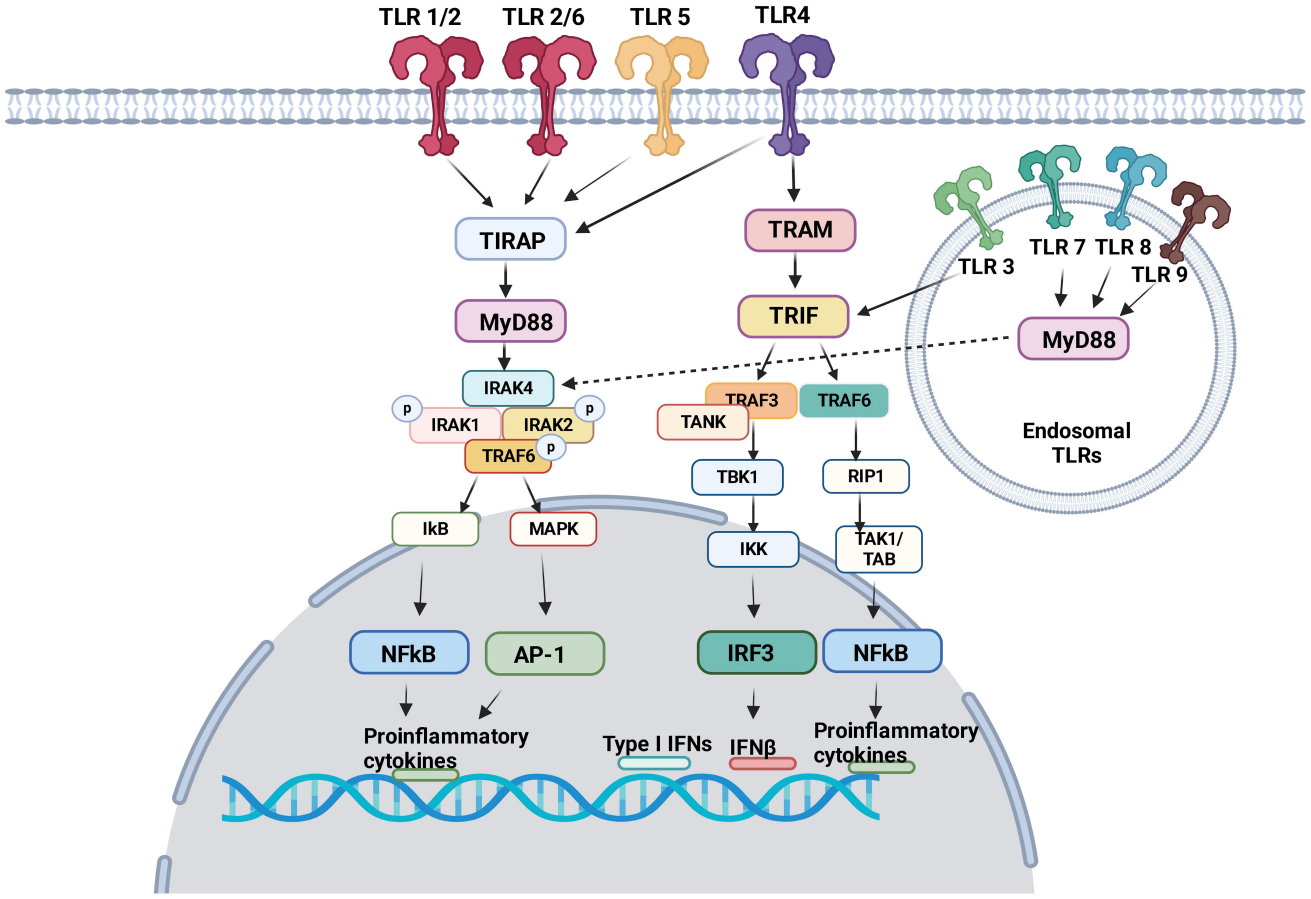
TLR signaling pathway TLR1, TLR2, TLR4, TLR5 and TLR6 are expressed on the cell surface. TLR3, TLR7, TLR8 and TLR9 are expressed in the endosome. Following ligand engagement, TLRs are dimerized, and interact either with MyD88 or TRIF. MyD88 signaling pathways involve NFκB and AP-1, both of which induces pro-inflammatory cytokines. Via TRIF, IRF3 and NFκB induces interferons and pro-inflammatory cytokines, respectively.

TLRs induce intracellular pro-inflammatory signaling events via myeloid differentiation primary response protein 88 (MyD88) and/or Toll/IL-1R (TIR) domain-containing adaptor inducing interferon (TRIF) (**Figure 1**). Here we focus on describing pro-inflammatory signaling pathways via MyD88 and/or TIR for TLR1–9. The dimerization of TLRs triggers signaling events.

### MyD88 signaling pathway

TIR domains are essential components of the innate immune system (36). The proximal events of TLR-mediated intracellular signaling are initiated by the interaction of TIR-domain of TLRs with TIR-domain-containing cytosolic adaptors and MyD88 is a central adaptor protein for TLRs. With the exception of TLR3, all TLRs mediate the downstream signaling pathway via MyD88 (37). The association of TLRs with MyD88 recruits the members of the interleukin-1 receptor associated kinase (IRAK) family, forming MyD88-IRAK-4 complex. This recruits IRAK-1 and IRAK-2, leading to the phosphorylation of IRAKs and interaction with tumor necrosis factor receptor associated factor 6 (TRAF6). TRAF6 induces the activation of transforming growth factor-β activated kinase 1 (TAK-1), thereby I-κB (IκB) and mitogen-activated protein kinase (MAPK). The activation of IκB and MAPK results in nuclear factor kappa B (NF-κB) and activator protein 1 (AP-1)-mediated gene transcription (38, 39). IRAK activation also stimulates interferon-regulatory factor (IRF) such as IRF7 (40–42) and activates the gene transcription of type I IFN (43). As a result, pro-inflammatory cytokines including tumor necrosis factor (TNF), interleukin (IL)-1, IL-6, IL-12, and interferon (IFN)-α are produced (44).

### TRIF signaling pathway

TRIF was identified as MyD88-independent pathway (alternative pathway). TRIF is recruited to TLR3 and TLR4. TRAF activation recruits TRAF6 and TRAF3. TRAF6 recruits receptor interacting protein 1 (RIP1). The subsequent activation of TGF-β-activated kinase 1 (TAK1)/TAK1-binding proteins (TABs) leads to the activation of NFκB and IFN-β promoter (45) to express pro-inflammatory cytokines and type I interferons. TRIF also activates TANK-binding kinase 1 (TBK1) and inhibitor of NF-κB kinase (IKK). Subsequently interferon regulatory factor 3 (IRF3) is activated and negatively regulates the activation of NF-κB and IFN-β promoter (46).

## Integrins

**Figure 2:**
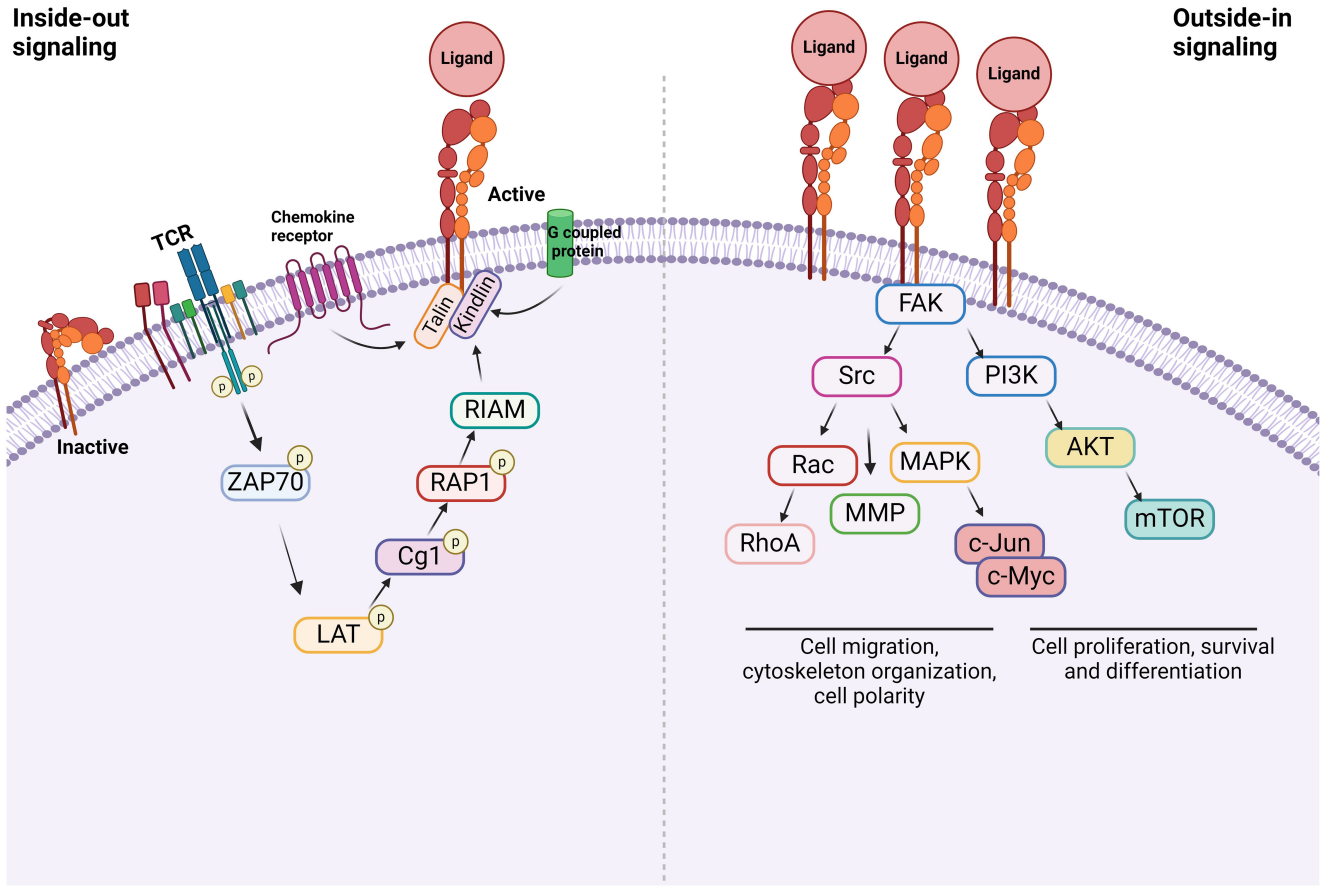
Integrin signaling Inside-out signal: Integrins are in an inactive conformation at baseline. However, the activation of receptors such as GPCRs, chemokine receptors, and TCR induces a cascade of events within the cells. The example shown here is via TCR. At the end, talin along with kindlin bind to β subunit of integrins, inducing its conformational change, which triggers the structural change of α subunit, allowing the integrin to bind to its ligand. Outside-in signal: Integrins that bind to their ligands cause cytoskeletal changes via focal adhesion molecules including focal adhesion kinase (FAK), leading to cell proliferation, survival, differentiation, and migration.

Integrins are α/β heterodimeric cell adhesion molecules that mediate cell-to-cell and cell-to-matrix interactions (47, 48). They are type I membrane glycoproteins with large extracellular domains, single transmembrane domains, and relatively short intracellular tails. The head of the large extracellular domain serves for ligand binding. To date, 18 α- and 8 β-subunits have been identified that combine to form at least 24 distinct α/β heterodimers. The list of integrins with representative ligands is included in **Table 2**. Integrins on the membrane (the outside) can receive signals triggered by non-integrin receptors via intracellular signaling (inside-out signaling) and vice versa (outside-in signaling) (**Figure 2**) (52).

**Table 2:** List of integrins, their ligands and expression distribution (48–51).

Integrins	Ligands	Expression
α1β1	Laminin, Collagen I, Collagen IV	Activated T and B cells, NK cell, glial cell, Schwann cells, fibroblasts, endothelium
α2β1	Laminin, Collagen, Tenascin	Activated T and B cells, NK cell, cell, fibroblasts, endothelium, epithelium
α3β1	Laminin, Fibronectin	Activated T cells, thymocytes, astrocytes, fibroblasts, endothelium, epithelium
α4β1	Fibronectin, VCAM-1, MAdCAM-1, TSP-1, Osteopontin,	T and B cells, NK cell, eosinophils, fibroblasts, endothelium
α5β1	Fibronectin, murine L1	Activated T and B cells, thymocytes, platelets, astrocytes, fibroblasts, endothelium, epithelium
αVβ1	Vitronectin, Fibronectin, Collagen, Fibrinogen, von Willebrand factor	Oligodendroglia
α6β1	Laminin	T cells, thymocytes, glial cell, fibroblasts, endothelium, epithelium
α7β1	Laminin	Melanoma, skeletal and cardiac muscle
α8β1	Fibronectin, Vitronectin, Tenascin	Neurons, oligodendroglia, epithelium
α9β1	Osteopontin, Tenascin, VCAM-1, Fibronectin, ADAM, VEGF	Epithelium (airway), muscle
α10β1	Collagen	Chondrocyte, mesenchymal stem cell
α11β1	Collagen	Skeletal and smooth muscle
αLβ2	ICAM-1, ICAM-2, ICAM-3	T cells, leucocytes, thymocytes,
αMβ2	ICAM-1, Factor X, iC3b, Fibrinogen	NK cell, activated B cell, myeloid cell, macrophage
αXβ2	iC3b, Fibrinogen	Activated B cell, myeloid cell, dendritic cell, macrophage
αDβ2	ICAM-1, ICAM-3, VCAM-1	Myeloid cell
αIIβ3	Fibronectin, Vitronectin, von Willebrand factor, Thrombospondin, Fibrinogen	Platelets
αVβ3	Fibronectin, Osteopontin, von Willebrand factor, PE-CAM-1, Vitronectin, human L1, Thrombospondin, Collagen	Activated T and B cells, monocytes, endothelium, glia
α6β4	Laminin	Schwann cell, endothelium, epithelium, fibroblasts
αVβ5	Vitronectin, Fibronectin, Fibrinogen	Monocytes, macrophages, oligodendroglia, epithelium, fibroblasts
αVβ6	Fibronectin	Epithelium
α4β7	Fibronectin, VCAM-1, MAdCAM-1	NK cell, T and B cell
αEβ7	E-cadherin	Intraepithelial T lymphocyte
αVβ8	Fibronectin, Vitronectin	Schwann cell, oligodendroglia, brain synapses

### Inside-out signal

Inside-out signal is initiated by non-integrin receptors such as G protein-coupled receptors (GPCRs), selectins, and chemokine receptors (49, 53, 54). Their signals are transmitted to activate integrins. Integrin αLβ2 was extensively studied on inside-out signal in the setting of T cells (55, 56). The activation of T cell receptor (TCR) and tyrosine kinase Lck leads to the phosphorylation of ZAP-70 kinase. This triggers the phosphorylation of LAT adaptor protein and the activation of phospholipase Cg1. This ultimately activates the small G protein ras-related protein-1 (Rap1). Rap1 binds to Rap-1 interacting adaptor molecule (RIAM). These events trigger the binding of talin and kindlin to β2 subunit, which induces the conformational change of αLβ2 into its active form (57). Although the binding of talin alone to integrin can activate it, its potency is extremely weak, supporting the critical role of kindlin in this process (58). The activation of αLβ2 results in its binding with ligands including intercellular adhesion molecule-1 (ICAM-1). What kind of molecules each integrin uses for inside-out signal and whether the same integrin uses different combination of molecules depending on cell type would be an important research area.

### Outside-in signal

Upon the inside-out activation, an integrin binds to a specific ligand. However, for the integrin to tightly bind to its ligand to mediate cell adhesion and migration, its cytoplasmic domains must be anchored to the cytoskeleton (59, 60). When the integrin binds to its ligand, it triggers the assembly of large protein complexes known as focal adhesions by incorporating a variety of molecules including cytoskeletal proteins and signaling molecules. Linking the integrin to the actin cytoskeleton promotes firm cell adhesion, cell spreading, migration and proliferation (57). Talin and kindlin serve as seed proteins to recruit proteins and initiate focal adhesion assembly (61). In case of αLβ2, the binding of ICAM-1 induces the activation/deactivation of kinases and phosphatases, leading to the cytoskeletal remodeling for the fine-tuning of effector functions such as T cell migration (62). Interestingly, this outside-in signal can be modified by the heterotrimeric guanine nucleotide-binding protein (G protein) Gα13. GPCRs activate Gα13, triggering its interaction with β integrin to regulate the outside-in signal (53).

## Integrin-TLR crosstalk

### β1 integrin

β1 integrin receptors regulate numerous functions, including cell adhesion, migration, differentiation, growth, and survival. β1 integrin subfamily consists of 12 α-chains that non-covalently bind to β1 chain (CD29) (49, 63). They can be categorized by their binding characteristics such as Arg-Gly-Asp (RGD)-binding integrins (αvβ1, α8β1, and α5β1), Leu-Asp-Val (LDV)-binding integrins (α4β1 and α9β1), collagen-binding integrins through triple helical GFOGER sequence in major collagens (α1β1, α2β1, α10β1, and α11β1), and laminin-binding integrins which includes both non-α Inserted (I) domain-containing integrins (α3β1, α6β1 and α7β1) and αI domain-containing integrins (α10β1, α2β1, and α1β1) (64). The key downstream signaling molecules of integrins include focal adhesion kinase (FAK), AKT, MAPK, Src-family protein tyrosine kinases, and integrin-linked kinase (ILK) (65). Integrins regulate intracellular signal transduction cascades that control differentiation, proliferation, and survival. Upon binding to fibronectin, collagen, and laminin, β1 integrin induces cell adhesion and migration that is extensively observed in pancreatic cancer models. Blockade or knockdown of β1 on cancer cells resulted better prognosis by reducing tumor growth and metastasis (66), which makes β1 integrin as an attractive therapeutic target. β1 integrins, in particular α9β1 has been reported to induce Th17 cell promoting cytokines in dendritic cells and macrophages in synergy with TLR2 and TLR4 through ERK pathway, that developed functional Th17 cells and arthritis (67). In addition to that, upon engaging with extracellular matrix (ECM) or other ligands, they initiate signaling pathways that can either reinforce or inhibit the activity of other receptors through negative or positive feedback loops. Interactions of α3β1 and α4β1 with TLRs have reported in several studies, which will be discussed in the following sections.

#### i. α3β1

α3β1 is expressed extensively on nearly all types of cells. It binds to a wide range of ligands with or without classical RGD integrin-binding motifs (68). α3β1 integrin serves as a receptor for collagen (type I and VI), laminin (α_1_β_1_γ_1_), laminin-5 (α_3_β_3_γ_2_), laminin-10 (α_5_β_1_γ_1_), laminin-11 (α_5_β_2_γ_1_), fibronectin, entactin, nidegon, and thrombospondin-1 with high specificities and affinities (69). Integrins are often targeted by bacterial and viral pathogens to adhere to and invade host cells. β1 integrins are particularly prone to their targets (70). β1 integrins serve as receptors for bacterial surface proteins including invasin and FimH (71, 72). α3β1 binds to BBB07 expressed on *Borrelia burgdorferi* (*B. burgdorferi*), the causative microbe of Lyme disease (73). BBB07 also serves as a TLR2 ligand. By ligation to the same ligand by both α3β1 and TLR2/1, human macrophages manifested enhanced pro-inflammatory responses to bacterial components.

α3β1 also mediates the endocytosis of TLR2 ligand Pam_3_CSK_4_, thereby facilitating its recognition by TLR2/1 within the endosome (74). This leads to the recruitment of adaptor molecules such as MyD88 by TLR2/1, eventually activating NF-κB signaling pathway and inducing the production of pro-inflammatory cytokines such as IL-6 (56). In murine macrophages, the endosomal activation of TLR2/1 induces IFN-β (75). This endocytosis mediated by α3β1 was observed for both live bacteria and bacterial proteins.

The impact on TLR2 mediated signaling via α3β1 is cell type-dependent (76). As in macrophages, α3β1 and TLR2/1 crosstalk selectively enhances IL-6 and IL-10 production by neutrophils in the setting of sepsis. However, neutrophils do not produce TNF production. Activated neutrophils release laminin (77) which bind to α3β1 on their cell surface, and increase the phosphorylation of FAK, but not Syk. This is responsible for the aforementioned profiles of pro-inflammatory cytokines by neutrophils (78). Activated FAK feeds into the MyD88-dependent TLR signaling. It is not certain about the presence of direct interaction between α3β1 and TLR2/1 on the neutrophils, but it is suggested that they may interact transiently within the lipid rafts upon activation since both of them localize there during activation (79, 80).

#### ii. α4β1

α4β1, also referred to as very late antigen-4 (VLA-4), is expressed on most leukocytes. It plays a crucial role in cell homing, trafficking, differentiation, activation, and survival. The ligands of this receptor include ECM protein fibronectin and the vascular cell adhesion molecule-1 (VCAM-1), which are expressed on endothelial cells (81). α4β1 binding site to fibronectin contains the tripeptide sequence Leu-Asp-Val (LDV) and is located in the alternatively spliced connecting segment 1 (CS-1) region, while VCAM-1 is recognized through the sequence Ile-Asp-Ser (IDS) (82). The domain called extra domain A (EDA) within fibronectin activates TLR4 (83). Thus, fibronectin severs as a ligand for both α4β1 and TLR4 (84). α4β1 was shown to function as a co-receptor for TLR4 in fibroblasts. Blockade of α4β1 or TLR4 or knockdown of α4 subunit in fibroblasts resulted in a decreased production of pro-inflammatory cytokines such as TNF and IL-10 (85).

### β2 integrin

β2 integrins consist of four members- αLβ2 (CD11a/CD18, lymphocyte function-associated antigen-1), αMβ2 (CD11b/CD18, macrophage-1 antigen, complement receptor 3), αXβ2 (CD11c/CD18, p150.95, complement receptor 4), and αDβ2 (CD11d/CD18). αLβ2 is ubiquitously expressed on all leukocytes, while αMβ2, αXβ2, and αDβ2 are mainly expressed on myeloid cells at different levels (86). αLβ2 binds to intercellular adhesion molecule (ICAM)-1~5 that can be found on the surface of other cells. αMβ2 has broad versatility, having over 40 known binding partners, such as ICAMs, iC3b, fibrinogen, RAGE (receptor for advanced glycation end products), and CD40L (87). αMβ2 and αXβ2 share several ligands as including iC3b, ICAM-1 and fibrinogen, but their binding sites on the same ligand are not exactly the same (88). αDβ2 also binds to multiple ligands, encompassing extracellular matrix-associated proteins like fibronectin, fibrinogen, vitronectin, and plasminogen as well as ICAM-1 (89). Reactive oxygen species (ROS) produced by the ligation of TLR2 and TLR5 induced rapid β2-integrin activation on myelomonocytes, and promoted leukocyte adhesion, suggesting that TLRs collaborate with one another (90). CD18 (β2) knockout (KO) macrophages and DCs produced higher level of IL-12p40 and IL-6 in response to TLR2, TLR4 and TLR9 stimulation, and higher level of type I interferon in response to TLR4 stimulation (91), suggesting that β2 integrins modulate TLR response. Further investigation of β2 ablation showed NF-κB and p38 MAPK pathway activations were involved in these processes (91, 92). Among β2 integrins, the interplay between αMβ2 and TLRs is well studied, which will be discussed further.

### i. αMβ2

αMβ2 is highly expressed on macrophages, DCs, monocytes, granulocytes, and mature or activated NK cells (93). It regulates TLR signaling positively or negatively, depending on cell types and inflammatory status.

**Figure 3:**
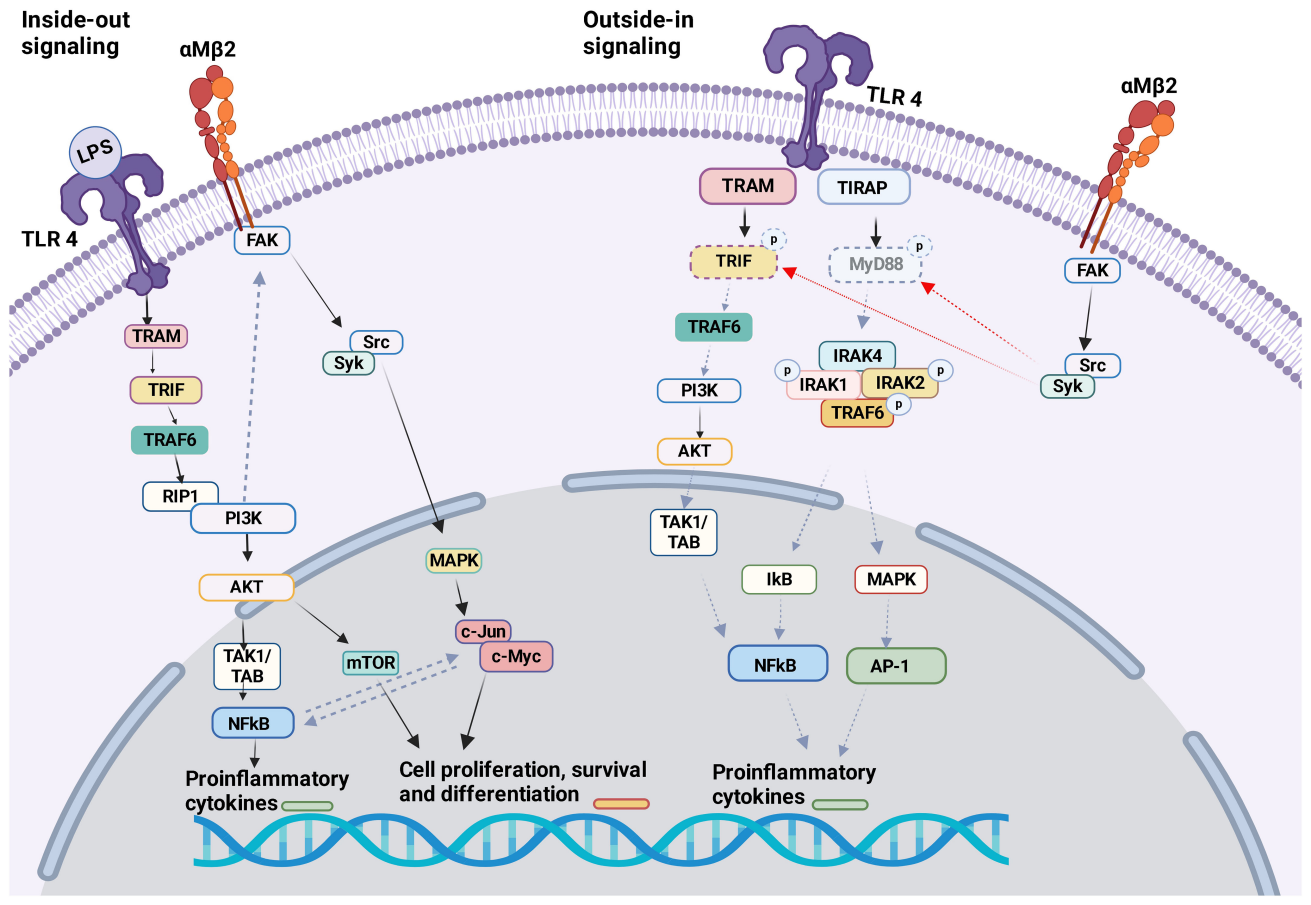
αMβ2 and TLR4 crosstalk The crosstalk between αMβ2 and TLR4 is shown.Inside-out signal: LPS binding to TLR4 induces the activation of many molecules including PI3K. PI3K facilitates the activation of αMβ2 intracellular adaptor proteins, therefore αMβ2 itself. Outside-in signal: Activated αMβ2 communicates with Src/Syk, which facilitates the degradation of MyD88 and TRIF. This will attenuate TLR4 activation signal. Of note, Syk is typically associated with other receptors like C-type lectin receptors (CLRs).

TLR4 KO neutrophils reduced αMβ2 activation, but not αLβ2 or αXβ2, suggesting that TLR4 would selectively facilitate the activation of αMβ2 on neutrophils. TLR4-mediated αMβ2 induction involved the activation of transcription factors NF-κB and c-Jun (94). αMβ2 can affect several TLRs. Upon *in vivo* challenge with TLR ligand stimulations (LPS, poly(I:C), and CpG) pro-inflammatory cytokines (TNF, IL-6, IL-10, and IFN-β) were greatly increased in the serum of CD11b (αM) KO mice (39). Higher level of pro-inflammatory cytokines in the serum was observed in CD11b KO mice during methicillin-resistant *Staphylococcus aureus* (MRSA) (95) and *Escherichia coli* (*E.coli*)<i> (96) </i>infection. Bacterial loads were higher in CD11b KO mice following MRSA and *E. coli* infections. In contrast, CD11b KO mice demonstrated better clearance of *L. monocytogenes* following its infection, despite higher serum TNF and IL-6 levels were detected (95). The difference in the phenotype may be because TNF induces apoptosis of certain bacteria (97). In case of MRSA and *E.coli* infection, TLR4 ligation activated αMβ2 on macrophages by inside-out signaling through PI3K and RapL pathway, which negatively looped back TLR4 signaling (**Figure 3**) (96). Outside-in signaling activated Src-Syk and promoted degradation of MyD88 and TRIF (**Figure 3**). This feedback loop in macrophages may control balance of both TLR4 and αMβ2 signaling pathways since their uncontrolled activation can cause harmful pathogenesis. Of note, syk is typically associated with other receptors such as C-type lectin receptors (CLRs). To make complicated further, resident macrophages or bone marrow derived macrophages from CD11b KO mice showed similar level of pro-inflammatory cytokines and activation status upon LPS stimulation, thus suggesting that the interplay of αMβ2 with TLR4 was not involved in steady-state macrophages (96). Thus, the interplay between TLRs and αMβ2 may be dictated by cell types and their cellular state. In fact, the lack of αMβ2 in DCs resulted in decreased pro-inflammatory cytokines and reduced MyD88-dependent phosphorylation of p38, Erk1/2, JNK, and IκBα in response to LPS stimulation (96). Upon stimulation with LPS, αMβ2 was clustered in DCs and co-localized with CD14, which has been shown important for TLR4 endocytosis, suggesting that αMβ2 was a part of TLR4 endocytosis. Furthermore, CD11b KO in DCs impaired RANTES production in LPS induced TRIF–mediated signaling in the endosome (44). Unlike TLR4, αMβ2 in DCs negatively regulated TLR9 signaling by selectively reducing IL-12p70 production, which was possibly regulated by upregulated miR-146. The consequence of IL-12p70 production affected poor cross-priming of DCs to cytotoxic T lymphocyte (CTL) response (98). TLR3 and αMβ2 interplay has been reported on NK cells. KO and neutralization of αMβ2 enhanced cytotoxic function of NK cells in response to TLR3 stimulation and limited acute liver infection (93). αMβ2 deficiency impaired the activation of MAPK/JNK pathway, suggesting that it inhibited TLR3 mediated activation of NK cells (99). Inside-out activation of αMβ2 by TLR2 in association with CD14 was reported in monocytes during the infection of *Porphyromonas gingivalis*, a pathogen implicated in chronic periodontitis and atherosclerosis. The activation of αMβ2 induced adhesion and recruitment of monocytes to the site of the infection (100). This recruited inflammatory monocytes can be beneficial to control infection, but uncontrolled accumulation results a tissue destruction. Although current data are all based on either αMβ2 or TLR KO system or depletion by neutralizing antibodies, the studies suggested a possible indirect interplay between αMβ2 and TLR2 (100).

While most studies examined the interaction between TLRs and αMβ2 by inhibiting or deleting αMβ2, some studied by activating it. αMβ2 activation by leukadherin-1 (LA1), its allosteric agonist, protected mice from pathological injuries and reduced the mortality induced by LPS (101). αMβ2 activation by LA-1 inhibited M1 macrophage response to LPS both *in vivo* and *in vitro*. Although it is not clear whether LA-1 facilitated a direct interaction between αMβ2 and TLR4 on macrophages, it induced an endocytosis of both αMβ2 and TLR4 and prevented LPS binding to TLR4 (101). While it prevented an excessive activation of TLR4 signaling pathway and pro-inflammatory response in macrophages, LA-1 pretreatment induced pro-inflammatory cytokines in DCs, suggesting that the effect of LA-1 could be cell type-dependent (101). It is worth noting that the expression levels of αMβ2 on macrophages and DCs are different (102), which may be in part responsible for the different effect of LA-1 on these two cell types.

A recent study showed that CD11b deficiency of donor non-classical monocytes increased CXCL2 production and exacerbated primary graft dysfunction in lung transplantation model (103). High mobility group box 1 (HMGB1), a DAMP released from dying cells, activates TLR4 in nonclassical monocytes. It was released from the donor lungs with primary graft dysfunction. Interestingly, HMGB1 stimulation induced lower CXCR2 production by TLR4 single KO or TLR2/TLR4 double KO monocytes, but not TLR2 single KO. It is uncertain whether αMβ2 interacts directly with TLR2 or TLR4, however, αMβ2 agonist LA-1 prevented primary graft dysfunction, suggesting that αMβ2 might facilitate TLR endocytosis.

Although all the evidence supports the presence of crosstalk between αMβ2 and TLRs, several questions still need to be answered. Do αMβ2 and TLRs bind to the same ligands? In fact, several studies reported that αMβ2 binds viral dsRNA (104), and bacterial toxins (105) and LPS (106–108). So far the interaction between ligands and αMβ2 has been shown *in vitro*. Therefore, it will be critical to determine if reported ligands for αMβ2 are in fact relevant *in vivo*. If so, it is not known whether both TLRs and αMβ2 bind a ligand at the same time. If they do, which signaling should be activated first? Does ligand binding avidity and affinity affect downstream signal? Or do they limit activation? If they don’t, is there ligand binding competition between TLR and αMβ2? Would it be possible that the rest of β2 integrin members interplay with TLRs? This may be very possible since extracellular part of αXβ2 has over 80% of sequence homology to αMβ2 and about 50% of homology in intracellular tail, for example (109, 110). Furthermore, LPS also binds to caspase-11 intracellularly (111), which makes the crosstalk complicated. β2 integrin members may also collaborate each other to inhibit or induce TLR response, since β2 integrin members are expressed upon activation of cells, especially on myeloid cells. They may function synergistically. Apparently, the proposed crosstalk between αMβ2 and TLRs depend on cell types, but does ligand binding affinity or avidity affect crosstalk? For example, high-affinity ligand binding affects the degree of up- or downstream signal? There might be a rivalry between the ligands. It is interesting to know whether these crosstalks depend on the timing of activation or not. A previous study showed αMβ2 on dendritic cells was activated through inside-out signaling by TLR4 (112) that was necessary for αMβ2-induced phagocytosis but not affected αXβ2, suggesting a bidirectional action between αMβ2 and TLR4.

### αV integrin

αV integrin also known as CD51 or MSK8, is a transmembrane protein that is involved in cell adhesion, migration, and signaling (113). αV integrin forms heterodimers with various β integrin subunits such as β1, β3, β5, β6, and β8. Together they designate a various array of receptors to bind to specific ligands in the extracellular matrix (ECM) including fibronectin, vitronectin, fibrinogen, and osteopontin, enabling cells to adhere and respond to their surrounding environment (114, 115).

In addition to adhesion, αV integrin promotes the activation of a multitude of signaling pathways, primarily the FAK pathway (116). The phosphorylation of FAK will in turn recruit Src kinases, phosphoinositide 3-kinase (PI3K) subunit p85, or phospholipase (PL)Cγ and stimulate the signaling cascades of Ras/Erk, PI3k/Akt, and Crk/Dock180/Rac. These pathways contribute to cell survival, proliferation, differentiation, and migration, emphasizing the multifaceted role of integrin αV.

The significance of αV integrin is not only linked with normal physiological functions. Dysregulation of αV integrin function has been associated with a variety of pathological conditions, including cancer, metastasis, angiogenesis, and wound healing (115). Additionally, αV integrin is involved in vascular remodeling and fibrosis (117).

#### i. αVβ3

αVβ3 is a multifaceted integrin due to its expression on a plethora of cell types and its ability to bind to many extracellular ligands. Through recognizing Arg-Gly-Asp (RGD) motif, αVβ3 binds to extracellular matrix proteins such as vitronectin, fibronectin, fibrinogen, and von Willebrand factor (48, 118). It can also serve as a receptor of some viruses for their entry into target cells (119). The αVβ3-TLRs cooperation has been described in several studies; Plasma membrane TLR4, TLR5 and endosomal TLR3 activated epithelial cells via NF-kB signaling pathway in response to viral and bacterial pathogen-associated molecular pattern molecules (PAMPs) (120). αVβ3 further enhanced their NF-κB activation. αVβ3 also positively orchestrated TLR2 signaling by facilitating a recruitment of the adaptor MyD88 to TLR2 (121). This mechanism was driven by a physical interaction of both αVβ3 and TLR2 with herpes simplex virus (HSV). This leads to NF-κB activation and the production of various mediators including IFN-α, IFN-β, IL-2, and IL-10 in response to the viral infection. Another type of αVβ3-TLR2 interplay has been attested in a different study, in which αVβ3 was shown serve as a co-sensor for bacterial lipopeptide (BLP) to be detected by TLR2 (122). The molecular mechanism mediating TLR2 activation was through the recognition of BLP by vitronectin on human monocytes. The TLR2-αVβ3 complex interaction was entirely dissociated following the completion of BLP stimulation. This further confirmed the physical link between αVβ3 and TLR2 in recognizing invading pathogens and initiating a synergistic response. The collaboration between αVβ3 and TLRs was also described in bacterial infection. In a murine cecal ligation and puncture (CLP)-induced sepsis and in a LPS-stimulated macrophage cell model, αVβ3 positively regulated TLR4 signaling in peritoneal macrophages (123). The deficiency of αVβ3 attenuated TLR4 activation. This effect appears to be mediated by CD14 expression, as αVβ3 deficiency inhibited CD14 expression. The deleterious impact of the αVβ3 -CD14-TLR4 crosstalk was caused by the release of a variety of pro-inflammatory cytokines. Therefore, CD61 (β3) KO mice exhibited higher survival rates and were more resistant to septic organ injury. A similar study revealed that thw previous crosstalk was mediated by WNT1 inducible secreted protein 1 (WISP1) (124). Ligation of WISP1 to αVβ3 synergistically enhanced TLR4-mediated TNF synthesis in LPS treated peritoneal macrophage.

#### ii. αVβ5

Similar to αVβ3, αVβ5 serves as a receptor for vitronectin (125). αVβ5 mediates phagocytosis of apoptotic cells and promotes angiogenesis and wound healing (126). The interaction of αVβ5 with TLR4 during infection was illustrated in a murine two hit-model of CLP and mechanical ventilation (MV)-induced lung injury (127). TLR4 KO mice showed better survival and less lung injury compared to wild type (WT) mice. αVβ5 regulated vascular permeability in both ventilator-induced lung injury (VILI) (128) and CLP (129). In line with this knowledge, neutralizing antibodies against αVβ5 partially attenuated lung injury. In this model, peritoneal macrophages increased the expression of αVβ5 in response to TLR4 activation. The connection between αVβ5 and TLR4 contributed to the exacerbations of the CLP-MV lung injury model.

#### iii. αVβ6

αVβ6 is expressed mainly on epithelial cells and involved in wound healing (130). Excessive production of αVβ6 leads to lung fibrosis and cancer (131). Activation of transforming growth factor-β1 (TGF-β1) represents the key role of αVβ6 (131, 132). In line with this, influenza infection stimulated TLR3 and further induced αVβ6-dependent TGF-β1 activation in epithelial cells (132). TLR3- αVβ6 crosstalk converged on the RhoA kinase that was activated by TLR3. RhoA kinase was further required to activate TGF-β1 via αVβ6. This suggests that the crosstalk was through a signaling pathway rather than a direct physical interaction between TLR3 and αVβ6. Blocking αVβ6 seemed to have no effect on the viral entry to the epithelial cells or the replication of viral genes. The biological consequences of TGF-β1 activation via αVβ6-TLR3 axis were epithelial cell death and accumulation of collagen in mouse lungs, which in turn promoted fibrosis. Another adverse effect of αVβ6 during influenza infection of lung epithelium was the suppression of type I IFN response (133). The IFN antiviral response was mainly mediated by endosomal TLR7. αVβ6 activated lysosomal autophagy machinery to remove TLR7, leading to the suppression of TLR7-mediated IFN signaling against Influenza infection. Opposite to αVβ3, αVβ6 seemed to have no physical interaction with TLRs.

## Conclusion

Without doubt, TLRs regulate major signaling pathways to modulate the degree of inflammation. While TLRs crosstalk is not exclusively restricted to integrins as complement system has been shown to intercommunicate with TLRs in the host immunity during infection (134), we highlighted ones involving integrins here. As there are a number of signaling pathways to regulate inflammation, it is not surprising that crosstalk system involving integrins has been established to coordinate inflammatory responses as we examined (135). Underhill has proposed several possibilities why the crosstalk has evolved; 1) To provide robust response against invading microbes. 2) Compensation against genetic diversity in host population, 3) Multiple receptors can facilitate a more tailored, specific response (136). The idea of “a more tailored, specific response” is very fascinating, because innate immune cells, which usually express TLRs predominantly, are rather considered promiscuous and relatively non-specific compared to adaptive immunity. Further understanding the role of crosstalks between TLRs and integrins would allow us to understand very complex system that innate immunity has developed and intervene if indicated.

## Author contributions

FA: Writing – original draft. GB: Writing – original draft. KY: Writing – original draft, Writing – review & editing.
